# Impact of electron-beam dose on microbial diversity and succession dynamics of refrigerated poultry meat during extended storage

**DOI:** 10.1016/j.psj.2026.107685

**Published:** 2026-08-22

**Authors:** Blesseth McDonald, Elena G. Olson, Pheron Collie, Olivia Hawkins, Gabriela K. Betancourt-Barszcz, Aaron R. Bodie

**Affiliations:** a University of Georgia, Athens, GA, USA; b University of Wisconsin-Madison, Madison, WI, USA; c Reveam, Inc. Norcross, GA, USA

**Keywords:** E-beam, Microbiome, Shelf-life, Storage, Tenderloins

## Abstract

Electron-beam (E-beam) irradiation is used as a post-harvest intervention to reduce microbial contamination in poultry products, yet less is known about how irradiation doses influence microbial succession during extended refrigerated storage. This study evaluated the effects of E-beam dose on the bacterial community structure of tray-packed chicken tenderloins stored under refrigeration for 27 days. Chicken tenderloins were treated with 0, 1.0, 1.5, 2.5, or 3.5 kGy and sampled on Days 3, 7, 24, and 27. Bacterial communities were characterized using 16S rRNA amplicon sequencing, and alpha diversity, beta diversity, and differential abundance analyses were used to assess dose-response and storage-associated changes. E-beam treatment and storage time interacted to significantly influence microbial community composition, as measured by Weighted UniFrac and Bray-Curtis distances (*P* = 0.038 and *P* = 0.05, respectively). Storage time was a major driver of succession, with significant differences in Shannon entropy (*P* = 0.03) and Faith’s phylogenetic diversity (*P* = 0.0001), along with changes in abundance-based community structure by Bray-Curtis dissimilarity (Q = 0.002). Weighted UniFrac analysis further showed significant differences across storage days, including Day 3 compared with Days 7, 24, and 27 (Q < 0.0061) and Day 7 compared with Days 24 and 27 (Q < 0.05). Taxonomic analysis displayed *Pseudomonas* increased throughout storage and approached approximately 80% relative abundance by the final sampling day (ANCOM, *P* < 0.05). Sequential ANCOM-BC comparisons further exhibited the enrichment of *Pseudomonas* from Day 3 to Day 7 (LFC = +3.52) and from Day 7 to Day 24 (LFC = +2.71). In contrast, several early-storage or competing taxa, including *Bacillus, Fabibacter, Yersinia, Carnobacterium, Aeromonas,* declined across later comparisons. E-beam dose also influenced community structure, with the highest dose (3.5 kGy) producing the most pronounced early shifts in microbial community composition. Overall, these findings suggest that E-beam irradiation shapes the initial poultry meat microbiome, while refrigerated storage remains a dominant factor influencing late-stage convergence toward a lower-diversity, *Pseudomonas*-dominated community.

## Introduction

The United States poultry industry plays a key role in global poultry production, consistently ranking among the world’s leading producers and distributors. Over the past several decades, the demand for chicken meat has increased substantially from ten (10) pounds per person in 1940s to most recently calculated consumption at sixty-eight (68) pounds per person in 2021 (USDA, 2023). In response to meeting this growing demand, the United States produced over forty-five billion pounds of meat in 2024 (USDA, 2025). This production volume places considerable pressure on industries to deliver safe and high quality products.

Despite advancements in processing, contamination by pathogenic and spoilage bacteria in chicken meat remains a challenge (S. H. Chen et al., 2020). These challenges are heightened by the extended production-to-consumption timeline for fresh poultry, contributing to significant food safety risks (Cerveny et al., 2009; Wardhana et al., 2021). Therefore, an effective post-harvest intervention is essential for controlling microbial populations and potentially extending the shelf life without compromising product quality (Bazaraa et al., 2025).

Electron beam (E-beam) irradiation has been shown to be an effective post-harvest technology capable of reducing microbial load on poultry meat (Lewis et al., 2002; Lung et al., 2015). Several studies have demonstrated a dose response relationship between E-beam irradiation and microbial reduction in meat products. Jangale et al. (2025) evaluated electron-beam doses ranging from 1 to 3 kiloGrays (kGy) in poultry and demonstrated that low-dose irradiation can effectively reduce surface contaminants without negatively affecting product quality. More recently, Kalapala et al. (2026) examined a broader dose range of 0.5 - 4 kGy. They found that 1 - 2 kGy substantially reduced microbial loads while 3 - 4 kGy consistently reduced counts below detection limits in both ground turkey and ground chicken. Indiarto et al. (2023) assessed higher doses from 3 to 7 kGy and concluded that irradiation at 4 kGy is the optimal dosage for maintaining consumer acceptable sensory attributes. Collectively, these studies demonstrate the initial bactericidal impact of E-beam application on poultry products.

While these studies establish the efficacy of E-beam irradiation, they provide little insight into how irradiation reshapes the surviving microbial community throughout refrigerated storage. Existing research focuses on short term bacteria reduction, and not longitudinal microbial ecology dynamics. As a result, strategies for shelf-life extension continue to rely largely on observation rather than predictive ecological understanding.

To address this gap, the present study examined the effect of E-beam doses of 0, 1.0, 1.5, 2.5, and 3.5 kGy on microbial community structure and succession in refrigerated poultry meat over a 27-day storage period, using 16S rRNA sequencing (Collie et al., 2026). By integrating dose-specific irradiation effects with ecological analysis of microbial succession, this study provides a more comprehensive understanding of how specific E-beam doses influence the microbial community dynamics, thereby providing insight into the optimization of E-beam irradiation for microbial intervention and poultry meat shelf-life management.

## Material and methods

### Microbial sampling

Boneless, skinless tray packed tenderloins (1.9-pound packs) were collected directly from a commercial poultry processing facility (USA). Samples were kept between 0 and 4°C until arrival at irradiation facility. Once samples arrived, they were passed through the E-beam system at Reveam, Inc. (Rio Grande Valley ECP Center), McAllen, Texas, USA, through their respective treatment groups: no-treatment control, 1 kGy, 1.5 kGy, 2.5 kGy and 3.5 kGy (Morehouse et al., 2018; Collie et al., 2026). These E-beam dosages were chosen for two reasons. Firstly, to stay under the maximum approved range of 4.5 kGy recommended by the United States Department of Agriculture-Food Safety and Inspection Service (1999).Secondly, to stay within acceptable quality parameters observed from previous research (Morehouse et al., 2018; Collie et al., 2026).

Following treatment, samples were shipped overnight to the University of Georgia department of Poultry Science, in Styrofoam trays, maintaining temperatures between 0 and 4°C. Upon arrival at the University of Georgia, all samples were placed in a 4°C walk-in cooler and kept until their respective sampling timepoint (3, 7, 24 and 27). Samples were stored through day 27 to capture a full trajectory of microbial succession beyond the typical refrigerated shelf life of poultry meat and evaluate the persistence of E-beam associated microbial effects during extended refrigerated storage.

**Fig. 1:**
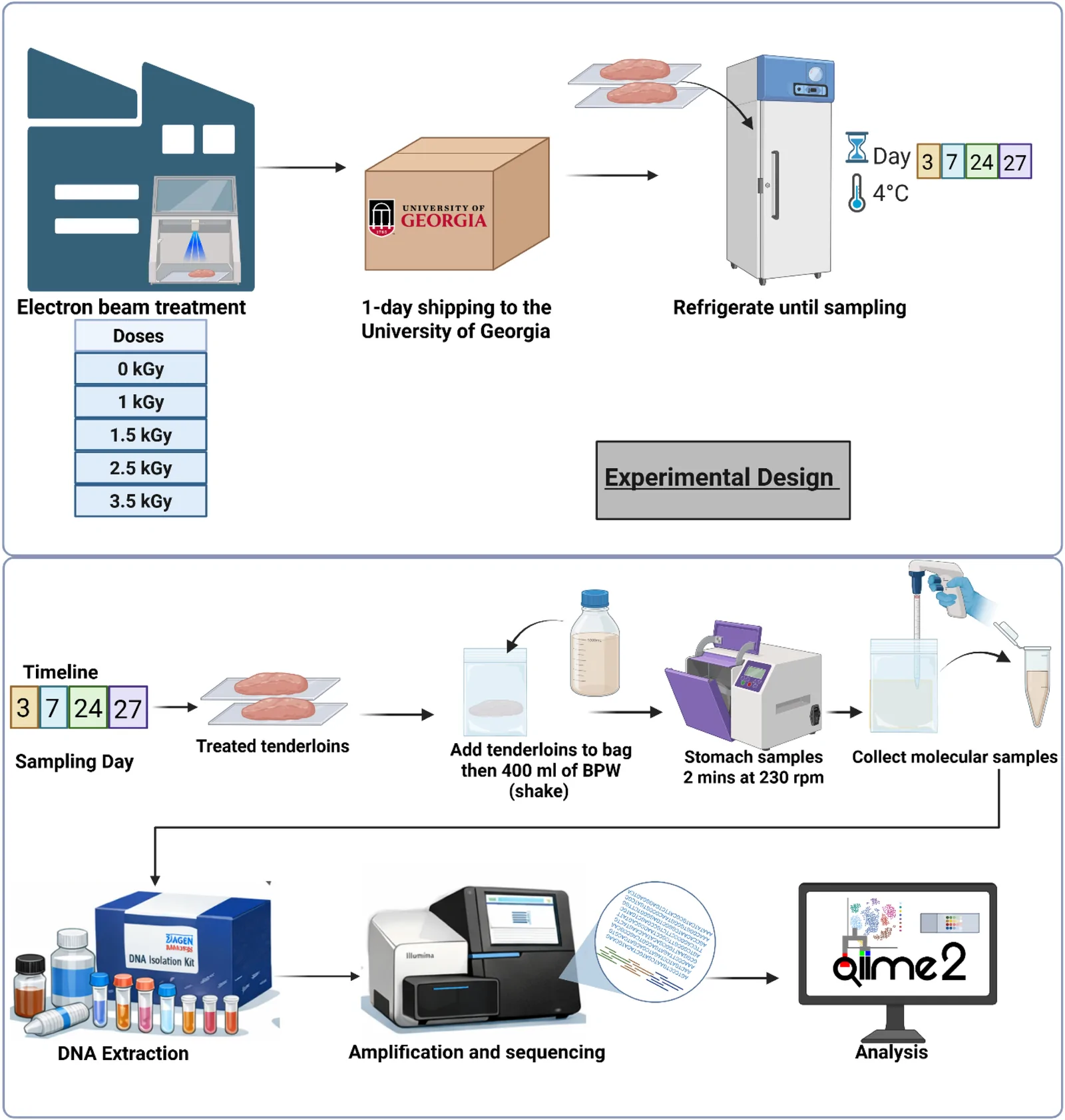
Experimental design for evaluating the effects of electron beam irradiation on chicken tenderloins across multiple doses and refrigerated storage duration. Samples were treated, shipped, stored at 4 °C, and sampled on days 3, 7, 24, and 27 for microbiome analysis. (Figure created using BioRender).

At each timepoint, the respective samples were removed from the 4°C refrigerator into the BSL2 laboratory of in the Poultry Science department at the University of Georgia. Tender samples were removed aseptically from the tray packs they were treated in and weighed up to 50 g. Next, tenders were added to Whirl-Pak bags (Whirl-Pak, Wisconsin, USA), where 400 mL of Buffered Peptone water (BPW) (Hardy Diagnostics, California, USA) was added, then stomached (Seward Stomacher Model 400; Seward Laboratory, New York, USA) for 2 minutes at 230 rpm. Homogenized sample rinsates were collected in 2.5 mL Eppendorf tubes (VWR, Radnor, PA) and frozen in liquid nitrogen, then stored at −80°C until further analysis (Figure 1).

### DNA extraction

Chicken tender homogenates collected on days 3, 7, 24, and 27 were thawed at room temperature. All samples were used less than 1 month after freezing. The genomic DNA from all samples was extracted using QIAGEN DNeasy Blood & Tissue Kit (Qiagen, Valencia, CA, USA). Once extracted, DNA samples were analyzed to determine their concentration and purity ratios (260/280, 260/230) using a Nanodrop Eight Spectrophotometer (Thermo scientifc, Waltham, MA, USA). All samples were either diluted or precipitated to have a final concentration of 10 ng/uL and stored at −20°C until the library preparation.

### Library preparation

An initial PCR was performed from DNA extracts using primers targeting the 16s: V4 genomic region using a BioRad T100 Thermal Cycler (Bio-Rad Laboratories, Hercules, CA, USA). The PCR was carried out using a KAPA HiFi HotStart ReadyMix PCR kit (REF KK2602) and thermocycler conditions were as follows: 95°C for three minutes, 30X cycles of 95°C for 30 s, 62°C for 30 s, and 72°C for 30, followed by a 72°C final extension for four minutes. PCR products were cleaned with a 1X volume bead clean-up using Omega BIO-TEK Mag-Bind TotalPure NGS clean-up beads (M1378-00). IDT NexTera adapters were added through a second PCR using the cleaned PCR products as template. This PCR was carried out using a KAPA HiFi HotStart ReadyMix PCR kit (REF KK2602) and thermocycler conditions were as follows: 95°C for three minutes, 18X cycles of 95°C for 30 s, 72°C for 30 s, and 72°C for 30, followed by a 72°C final extension for four minutes. Final PCR products were clean like the previous PCR products.

Libraries were equimolarly pooled based on DNA concentrations determined through photospectropic measures of SYBR green and with average fragment size quantified with an Advanced Analytical Fragment Analyzer. The final pool was quantified with Qubit version 4 and using qPCR (KAPA Library Quant Kit, REF 07960298001), which was performed on a Roche LightCycler 480 II. Analysis of fluorescent data was performed in the LightCycler 480 software version 1.5.1.62 SP3, and the distribution of DNA fragment sizes was quantified with an Advanced Analytical Fragment Analyzer. The pooled libraries were sequenced on an Illumina MiSeq using an Illumina MiSeq V2_500 cycles reagent kit, which produces 250 bp paired end reads. Amplicon library construction and sequencing were performed at the University of Georgia's Georgia Genomics and Bioinformatics Core, GGBC, UG Athens, GA, RRID:SCR_010994. The sequences were uploaded to BaseSpace (Illumina, San Diego, California, USA), the NCBI Sequence Read Archive (PRJNA1473010), and GitHub (https://github.com/BodieLab/E-beamPoultry/).

### Bioinformatic analysis

The sequenced products were analyzed using Quantitative Insights into Microbial Ecology 2 (QIIME2) pipeline, version 2025.10 (Bolyen et al., 2019). Demultiplexed sequencing reads were downloaded as FASTQ files from Illumina’s Base space website. Using the QIIME 2 pipeline, sequenced reads were imported using single lane paired-end demultiplexed format via qiime tools import, then filtered and denoised in DADA2 (Callahan et al., 2016) using q2-dada2 and generated Amplicon Sequence Variant (ASV). ASV’s were used for microbial community analyses. Taxonomic identities were assigned to representative ASV sequences using the classify-sklearn method implemented in q2-feature-classifier against the SILVA version 138.1 reference database.

### Statistical analysis

Q2-diversity feature was used to evaluate Alpha (α) and beta (β) diversity (Whittaker, 1972). The main effects and interactions for α-diversity were examined using ANOVA via q2-longitudinal. For β-diversity, interactions were assessed using permutational multivariate analysis of variance (ADONIS; To uncover α-diversity metrics the Faith’s Phylogenetic Diversity and Shannon’s Entropy (Shannon, 1948; Faith, 1992) were examined. The Kruskal–Wallis test was used for pairwise comparison. When analyzing β-diversity metrics comparisons, Bray-Curtis, Weighted Unifrac and Unweighted UniFrac were examined (Bray and Curtis, 1957; Lozupone et al., 2007). Using analysis of similarity (ANOSIM) mean population variation and dispersion, including Significant features were graphically represented through Emperor Principal Coordinates Analysis (PCoA) plots. Lastly, taxonomic variance abundance associated with each day was analyzed using ANCOM via q2-composition at the genus level. ANCOM tables were imagined in Microsoft Excel (Microsoft, Redmond, WA, United States). When generating relative abundance taxa bar plots, any taxa not in the top 17 abundance were categorized as "other". Main effects and pairwise differences were significant at *P* ≤ 0.05 and Q ≤ 0.05, respectively.

## Results

In the current study, 16S rRNA amplicon sequencing was used to evaluate how E-beam irradiation dose and refrigerated storage shaped bacterial communities associated with tray-packed chicken tenderloins. Samples were compared across five treatment groups, including the untreated control, 1 kGy, 1.5 kGy, 2.5 kGy, and 3.5 kGy, and across four refrigerated storage timepoints: Day 3, Day 7, Day 24, and Day 27. Together, the results indicate that E-beam irradiation influenced early microbial community structure, while storage time drove continued microbial succession and late-stage community convergence.

### E-beam dose influenced the trajectory of microbial succession during refrigerated storage

The interaction between E-beam treatment and storage day significantly influenced microbial community composition on chicken tenderloins. The treatment × day interaction significantly modulated both abundance and phylogenetic structure of microbial communities (Weighted UniFrac, *P* = 0.038; Bray-Curtis, *P* = 0.05; Supplementary Table 5). Shannon entropy was also significantly modulated over time, indicating that within-sample diversity and evenness changed during storage (*P* = 0.006; Supplementary Table 1). Overall, the interaction effect indicates that irradiation influenced the direction and magnitude of change across storage rather than producing static compositional differences.

### Refrigerated storage drove progressive changes in microbial diversity and community structure

**Fig. 2:**
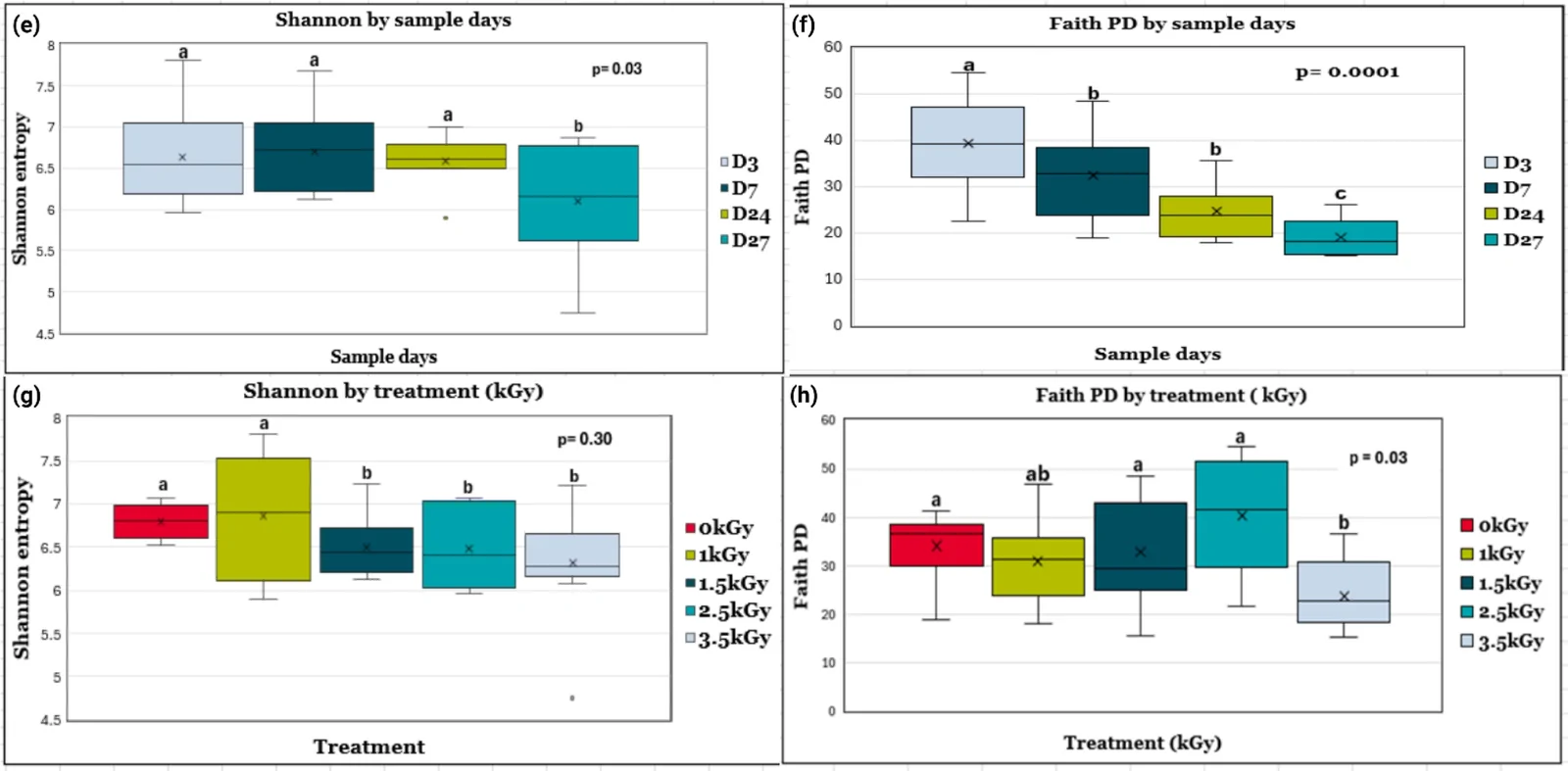
Alpha diversity metrics e Shannon entropy by sample days f Faith’s phylogenetic diversity by sample days g Shannon entropy by treatment h Faith phylogenetic diversity by treatment. Comparing the microbial diversity of the samples collected across the various treatments and days. The median value is shown as a horizontal line within the boxes. The inter-quartile range from lower to upper quartile represents the middle 50% of values for each group. The p-values were calculated using Kruskal–Wallis test. (Figure generated using Excel and BioRender).

Storage time had a strong effect on microbial diversity and community composition. Shannon entropy differed significantly by storage day (*P* = 0.03; Supplementary Table 1), although no pairwise comparisons between individual days remained significant after multiple-comparison correction (Q > 0.05; Supplementary Table 2). In contrast, Faith’s phylogenetic diversity showed a strong day effect (*P* = 0.0001; Fig. 3a), with Day 3 having the highest phylogenetic diversity compared with later storage timepoints. Beta diversity analyses further demonstrated storage-driven restructuring of the microbial community. Day 3 communities differed significantly from Day 7 and Day 24 communities (Bray-Curtis, Q = 0.002; Fig. 3; Supplementary Table 6), indicating a shift in abundance-based community composition during storage. Phylogenetic composition on Day 3 differed significantly from Day 7, Day 24, and Day 27 (Weighted UniFrac, Q < 0.0061; Supplementary Table 8), and Day 7 differed significantly from both Day 24 and Day 27 (Q < 0.05; Fig. 2; Supplementary Table 8). These results suggest that refrigerated storage influenced both the abundance and phylogenetic structure of the bacterial community.

Taxonomic analysis supported this storage-driven succession pattern. *Pseudomonas* increased throughout storage and reached nearly 80% relative abundance by the final sampling day (ANCOM, *P* < 0.05; Fig. 5). In contrast, *Bacillus* relative abundance was significantly reduced by Day 24. Sequential ANCOM-BC comparisons further indicated that *Pseudomonas* was enriched from Day 3 to Day 7, showing a log-fold change of +3.52. From Day 7 to Day 24, *Pseudomonas* remained enriched (LFC = +2.71), while *Bacillus* and *Soonwooa* were among the most depleted taxa. From Day 24 to Day 27, *Carnobacterium* (LFC = +1.91) and *Yersinia* (LFC = +1.54) showed modest enrichment, while *Fabibacter* (LFC = −1.72) and *Bacillus* (LFC = −1.48) were depleted. Together, these results indicate that storage time was associated with a progressive reduction in microbial diversity and selected for a narrow community dominated by storage-associated taxa, particularly *Pseudomonas*.

### Higher E-beam doses were associated with more pronounced shifts in phylogenetic diversity

E-beam irradiation influenced microbial diversity and community structure, with the most substantial treatment-associated differences observed at the highest dose. Shannon entropy showed trending pairwise differences between 0 kGy and 3.5 kGy and between 2.5 kGy and 3.5 kGy (*P* = 0.01; Q = 0.07 for both comparisons; Supplementary Table 6). Faith’s phylogenetic diversity differed significantly by treatment (*P* = 0.003; Fig. 2; Supplementary Table 1). Pairwise comparisons showed that the 3.5 kGy treatment differed from the untreated control (*P* = 0.01; Q = 0.07) and from the 2.5 kGy treatment (*P* = 0.01; Q = 0.07; Fig. 2(h); Supplementary Table 3). Treatment associated differences were also reflected in beta diversity. Treatment-related differences between the 2.5 kGy and 3.5 kGy groups were observed (Weighted UniFrac, *P* = 0.01; Q = 0.10; Fig. 3(b); Supplemental Table 7), indicating that the highest irradiation dose produced a distinct phylogenetic community structure compared with the lower dose.

Differential abundance analysis further supported this treatment-associated restructuring. A total of 122 taxa were significantly differentiated between the 2.5 kGy and 3.5 kGy treatments. Of these, 69 taxa were enriched in the 3.5 kGy group compared with the 2.5 kGy group, including *Comamonas* (LFC = +1.54), *NS9 marine group* (LFC = +1.53), *Fabibacter* (LFC = +1.52), and *Chryseobacterium* (LFC = +1.41). In contrast, 53 taxa were depleted in the 3.5 kGy group, including *Spiroplasma* (LFC = −1.56), *Brochothrix* (LFC = −1.53), and *Thalassolituus* (LFC = −1.41). Across all treatments relative to the untreated control, E-beam irradiation produced consistent directional shifts in community composition (Fig. 5). These findings suggest that the highest E-beam dose was associated with the greatest shifts in microbial community structure. However, since several pairwise comparisons did not remain significant after multiple comparison correction. Therefore, these findings should be interpreted as dose-associated changes in community structure rather than a consistent linear dose-response pattern (Fig. 7).

### Taxonomic succession differed by dose early in storage but became more consistent by late storage

To determine when irradiation effects emerged during storage, microbial diversity and taxonomic composition were compared among treatments within each sampling day. On Day 3, phylogenetic diversity showed trending pairwise differences between 0 kGy and 3.5 kGy and between 2.5 kGy and 3.5 kGy (Faith PD, *P* = 0.04 for both comparisons; Fig. 2h; Supplemental Table 4). These results suggest that the highest E-beam dose produced detectable early changes in phylogenetic diversity. By Day 27, all E-beam treatments showed a trend toward differentiation from the untreated control (Faith PD, *P* = 0.04; Q = 0.12; Fig. 2f; Supplemental Table 4), suggesting that treatment-associated differences persisted or re-emerged after extended refrigerated storage.

Taxonomic analysis showed that early irradiation effects were broad and dose-specific. On Day 3, E-beam treatment produced a wide range of compositional differences across doses, with each treatment showing a distinct pattern of enriched and depleted taxa. In the 1 kGy group, 89 taxa were enriched, and 68 were depleted compared with the untreated control. *Bradyrhizobium* was the most enriched taxon (LFC = +4.68), while *NS4 marine group* was the most depleted taxon (LFC = −3.26). The 1.5 kGy and 2.5 kGy treatments also showed broad restructuring, with enriched taxa including *SAR92 clade* (LFC = +3.48), *Polaribacter* (LFC = +2.92), and *Spiroplasma* (LFC = +2.92). At the highest dose, 3.5 kGy, 78 taxa were enriched, and 79 were depleted, with *Altererythrobacter* (LFC = −4.60), *Serratia* (LFC = −4.24), and *Subgroup 2* (LFC = −4.21) among the most strongly depleted taxa. By Day 7, treatment effects remained detectable but were less distinct across doses. Across all E-beam treatments, enriched taxa were dominated by *Afipia*, with LFC values ranging from +4.55 to +3.11, along with several uncultured groups. Depleted taxa consistently included *Stenotrophomonas* (LFC = −3.25) and *Nitrosotalea* (LFC = −2.81). Although LFC values remained high, greater overlap in enriched and depleted taxa across treatments suggested early convergence of microbial communities after the initial irradiation-associated disruption. By Day 24, a more consistent ecological pattern emerged across irradiation treatments. The number of differentially abundant taxa decreased across most treatment groups, with 61 differentiated taxa in the 1 kGy group, 48 in the 1.5 kGy group, 59 in the 2.5 kGy group, and 44 in the 3.5 kGy group (Supplemental Figs. 9–12). Across all E-beam doses, commonly enriched taxa included *Bacillus* (LFC = +4.83), *Alcanivorax* (LFC = +4.69), and *Rhodobacter* (LFC = +3.67). Depleted taxa also followed a more consistent pattern across treatments, with *Yersinia* (LFC = −4.83), *Carnobacterium* (LFC = −3.89), *Aeromonas* (LFC = −3.80), and *Shewanella* (LFC = −4.99) among the strongest depletions. By Day 27, the total number of differentially abundant genera continued to decrease in most treatment groups, with 31 differentiated taxa in the 1 kGy group, 23 in the 1.5 kGy group, 20 in the 2.5 kGy group, and 47 in the 3.5 kGy group. *Unassigned* taxa represented the largest enriched category across treatment groups. Depletion patterns remained similar to Day 24, with *Yersinia* and *Brochothrix* among the most consistently depleted taxa across treatments. Ultimately, these results suggest that E-beam irradiation was associated with early dose-specific restructuring of the poultry meat microbiome. However, as refrigerated storage progressed, treatment-specific differences became less distinct, and communities showed evidence of convergence toward a narrower late-storage microbiome.

## Discussion

### E-beam irradiation is associated with early changes in microbial community structure, while refrigerated storage drives later community selection

The results of the current study show that electron-beam irradiation altered the microbial ecology of tray-packed chicken tenderloins during refrigerated storage. Importantly, the effect of E-beam treatment was not limited to a single immediate reduction in microbial diversity. The significant treatment × day interaction observed for phylogenetic and abundance distances indicates that irradiation changed the trajectory of microbial succession over time. These findings suggest that E-beam treatment acts as an ecological disturbance that reshapes the initial microbial community and influences which bacterial populations survive, decline, or proliferate during storage. E-beam irradiated doses evaluated in the current study were below the 4.5 kGy maximum specified for uncooked meat and poultry products (Federal Register, 2012). Irradiation studies often emphasize microbial reduction, pathogen inactivation, or shelf-life extension (Lewis et al., 2002; Lim et al., 2008; Song et al., 2009; Indiarto et al., 2023). Previous research showed that E-beam and other irradiation technologies can reduce initial microbial loads during storage in meat products (Lim et al., 2008; Song et al., 2009). For example, Lewis et al. (2002) reported that E-beam irradiation reduced aerobic bacterial load and eliminated *Salmonella, Campylobacter*, and general *Escherichia coli* from boneless skinless chicken breasts. Similarly, Kalapala et al. (2026) explained that E-beam technology can effectively reduce foodborne pathogens and may potentially increase the shelf life of meat products at specific doses.

The current study adds a microbial ecology perspective by showing that E-beam dose can also influence community succession. Irradiation may reduce the number of microorganisms present after treatment while shaping the subsequent dynamics of the surviving organisms during refrigerated storage. Presently, 3.5 kGy produced the most pronounced phylogenetic shift (Figure 2h). This suggests that higher-dose E-beam treatment may have a greater influence on the composition and diversity of the bacterial community, resulting in more pronounced early shifts in community structure. However, because several pairwise comparisons showed trends after multiple-comparison correction, these results should be interpreted as evidence of biologically meaningful dose-associated shifts rather than a fully linear dose response relationship.

Although E-beam dose influenced early microbial community structure, refrigerated storage time emerged as the primary factor shaping microbial succession over the longer storage period. Across the 27-day storage period, communities showed changes in diversity, phylogenetic composition, and abundance-based community structure (Figs. 2–3a& 3b). This pattern suggests that storage progressively influenced the composition of the surviving microbial community, favoring microorganisms capable of surviving and proliferating under cold, packaged storage conditions. This finding is consistent with the broader ecology of chilled poultry and meat products. Fresh poultry is highly perishable, and USDA guidance recommends using raw poultry within 1 to 2 days when stored under home refrigeration conditions (Kosa et al., 2019; USDA, 2023). Under extended refrigerated storage, psychrotrophic spoilage organisms can become dominant, particularly when competing taxa decline or when storage conditions favor cold-tolerant aerobic or facultative organisms (Luong et al., 2020). In the current study, changes in phylogenetic diversity and community structure support the ecological narrowing interpretation. The community observed on Day 3 was more phylogenetically diverse and compositionally distinct from those observed at later timepoints, whereas the Day 24 and Day 27 communities reflected the cumulative influence of the extended refrigerated storage. These results suggest that E-beam irradiation helps shape the initial microbial community, while storage conditions subsequently influence the composition and relative abundance of populations throughout shelf life.

**Fig. 3a:**
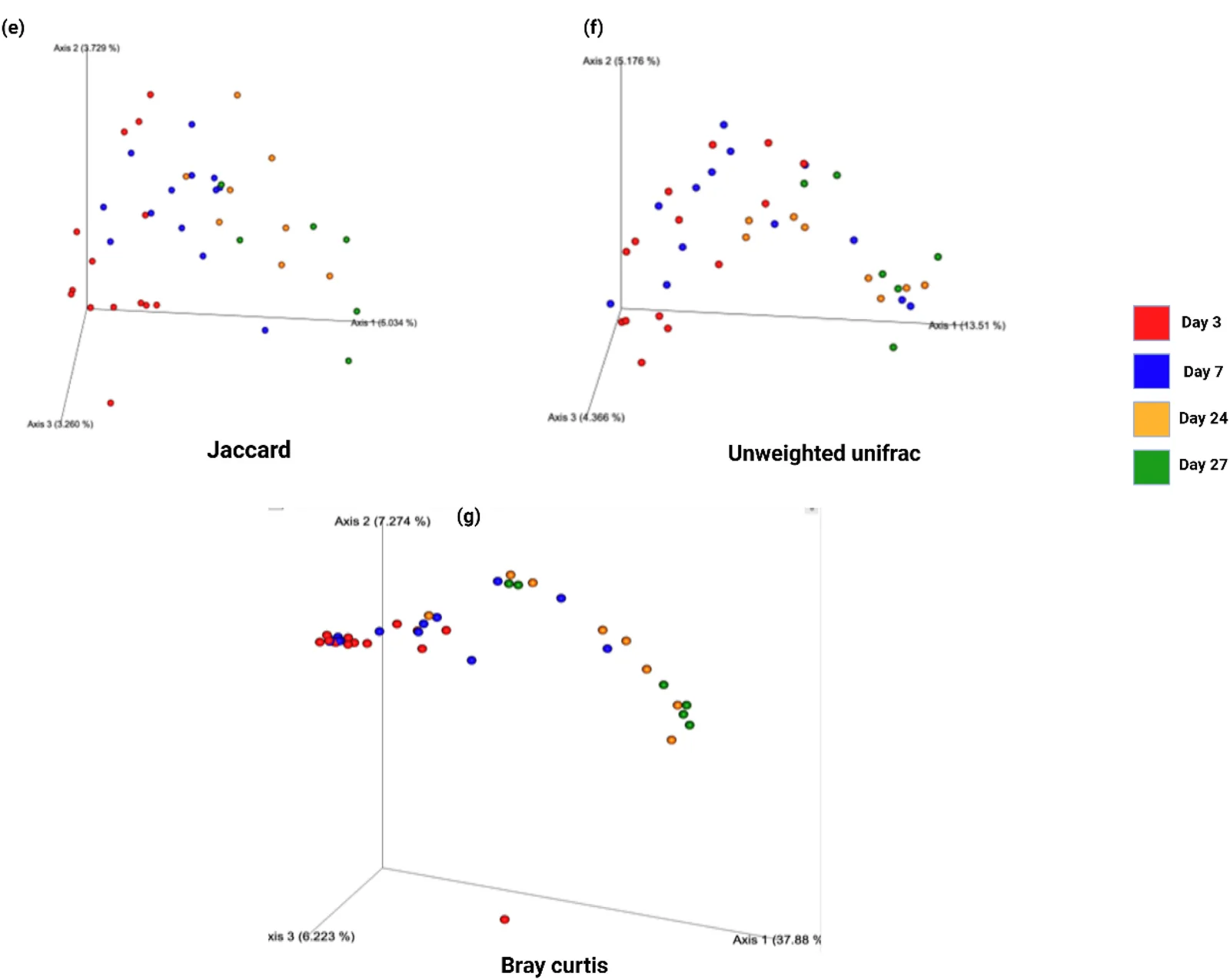
Principal coordinates analysis PCoA of samples across refrigerated storage using e Jaccard, f Weighted unifrac, and g Bray–Curtis distance matrixes. Each Points represent individual samples colored by storage day 3, 7, 24, 27. Axes indicate the percentage of variance explained for each ordination dimension. (Figure generated using QIIME 2 and BioRender).

**Fig. 3b:**
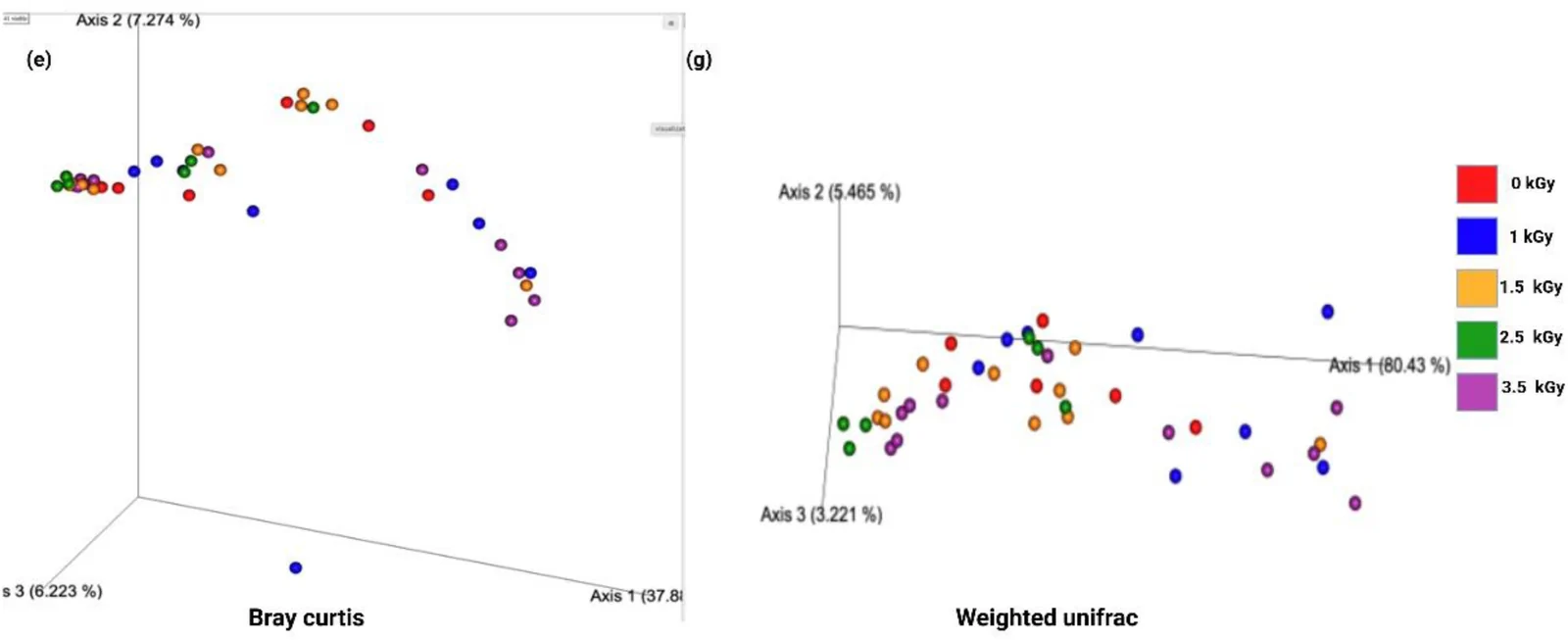
Principal coordinates analysis PCoA of samples across treatments, e Bray–Curtis and g weighted unifrac distance matrixes. Each Points represent individual samples colored by storage day 3, 7, 24, 27. Axes indicate the percentage of variance explained for each ordination dimension. (Figure generated using QIIME 2 and BioRender).

### *Pseudomonas* dominance marks late-storage convergence following dose-specific early shifts

**Fig. 4:**
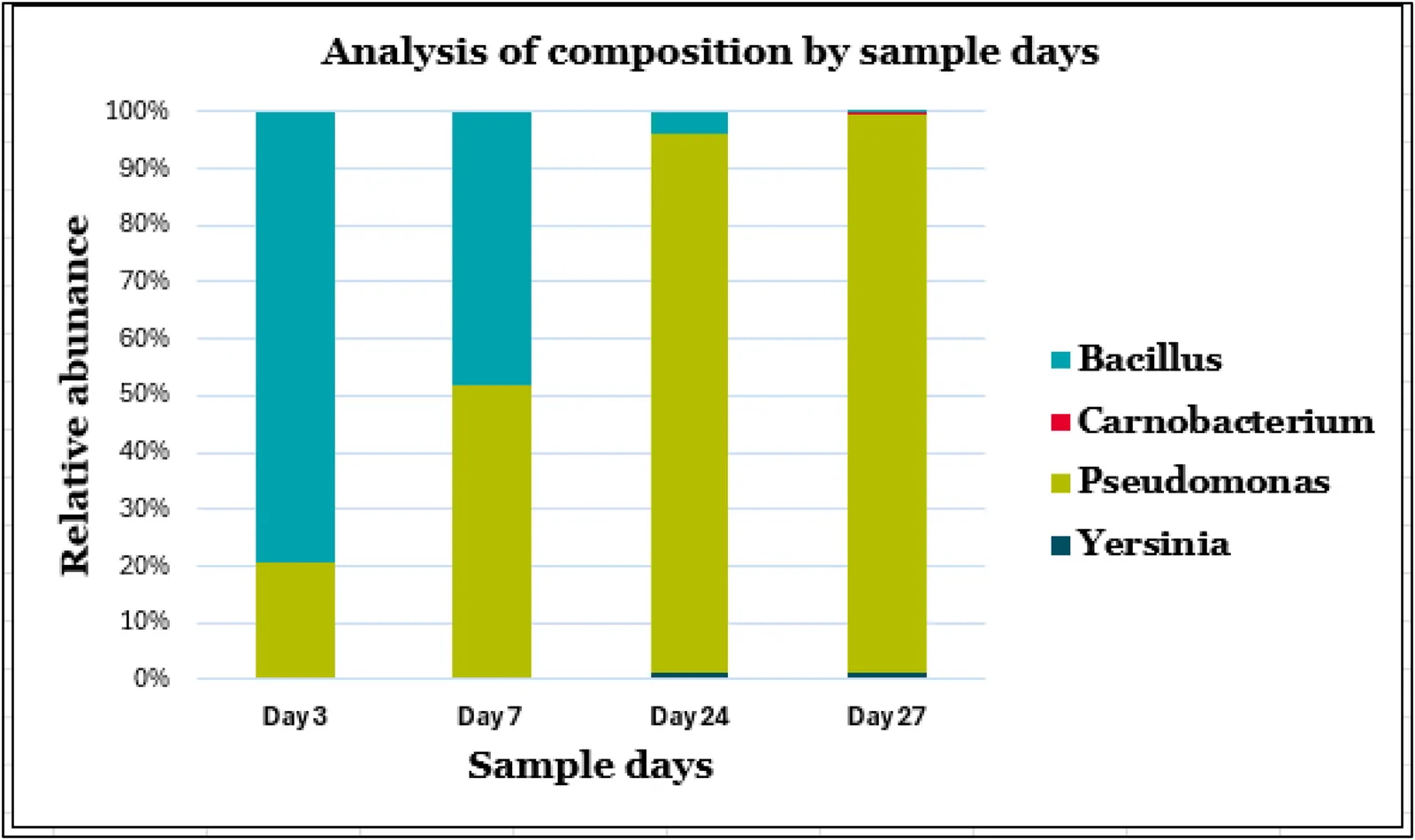
Relative abundance depicts storage-associated *Pseudomonas* dominance. Bars represent microbial community on the genus level for each sample day. (Figure created using Excel).

The most prominent taxonomic change observed during storage was the increase in *Pseudomonas*, which approached approximately 80% relative abundance by the final sampling day (Fig. 6). Sequential ANCOM-BC comparisons further showed enrichment of *Pseudomonas* from Day 3 to Day 7, with *Pseudomonas* remaining enriched from Day 7 to Day 24 (Fig. 4). These results suggest that *Pseudomonas* became a major contributor to late-storage community composition. This pattern is biologically consistent with the ecology of poultry spoilage. *Pseudomonas* genera are commonly associated with the spoilage of chilled poultry and other fresh meats, especially under refrigerated aerobic storage conditions (Dourou et al., 2021; Wang et al., 2017; W. Chen et al., 2017). *Pseudomonas*, in particular, has been identified as an important spoilage-associated organism in chilled chicken, and recent work suggests that *Pseudomonas* can influence microbial community structure and meat quality through metabolic activity (De Filippis et al., 2018).

The observed enrichment of *Pseudomonas* during storage may reflect its greater ability to persist and proliferate under refrigeration after treatment than more sensitive community members. E-beam treatment appears to reduce the abundance of susceptible microorganisms, influencing the initial community structure and the relative representation of more resilient taxa such as *Pseudomonas*. Refrigerated storage favored psychrotrophic taxa regardless of initial treatment. This pattern aligns with competitive dynamics. Microorganisms that persist after treatment suppress slow-growing organisms by competitive advantage during storage as they utilize available resources (France and Remold, 2016). Thus, E-beam reduces the susceptible microbial population and shapes the initial community structure. Refrigerated storage then selects for psychrotrophic organisms, enabling *Pseudomonas* and other cold-adapted spoilage organisms, such as *Acinetobacter* (Fig. 4)*,* to become dominant as storage time becomes the primary ecological driver. These interpretations suggest a succession-based explanation rather than a simple presence-or-absence effect.

**Fig. 5:**
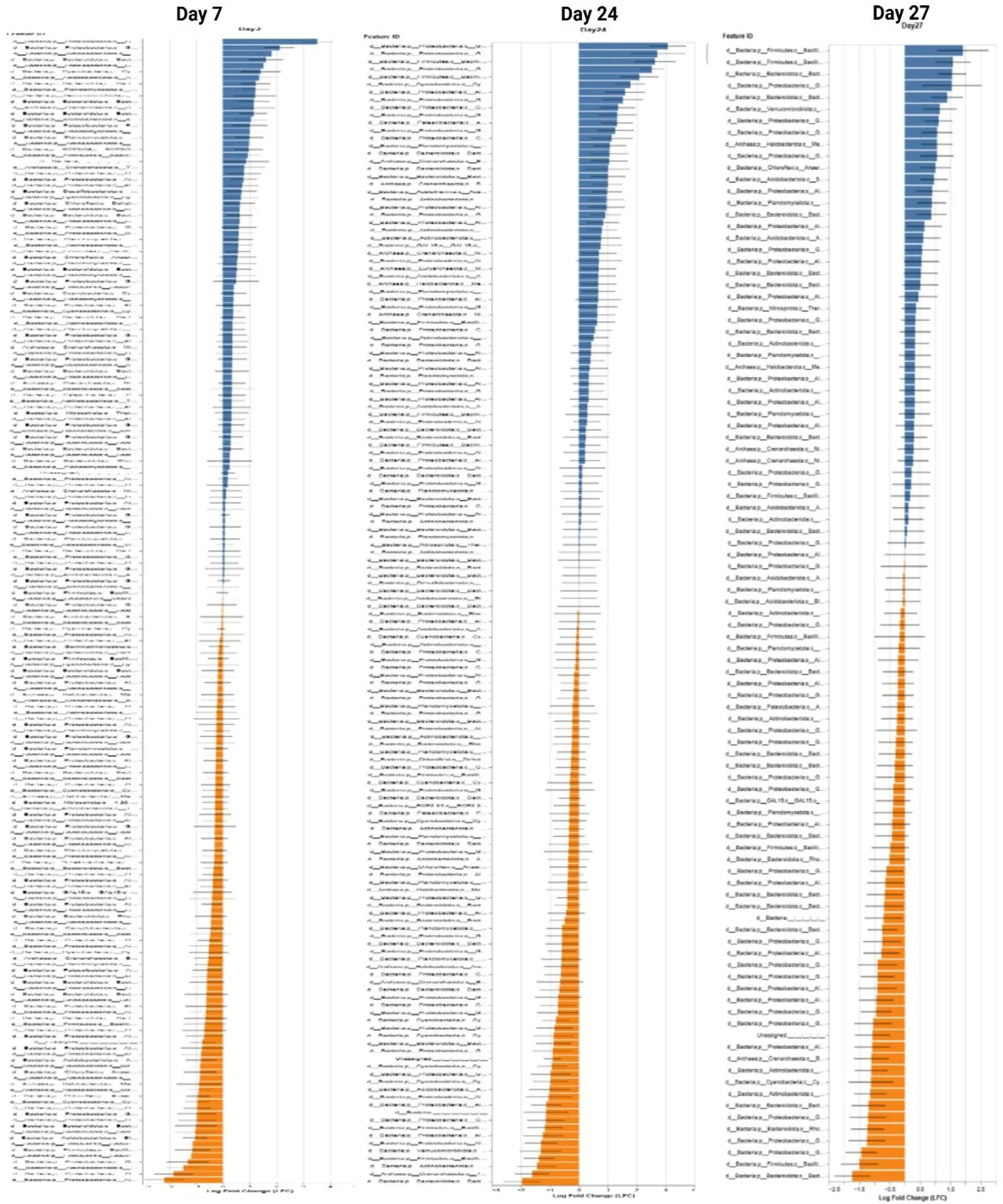
Differential abundance during storage for Day 7, 24 & 27 showing enrichment and depletion patterns (Figure generated using QIIME2 and Bio Render).

The treatment-level results suggest that 3.5 kGy produced the most pronounced changes in microbial community structure, especially in phylogenetic diversity (Fig. 2h). However, the day-specific taxonomic results also suggest that the microbial communities converge during extended storage. On Day 3, each E-beam dose exhibited a distinct taxonomic profile, with different enriched and depleted genera across treatments. By Day 7, treatment associated effects were still present but less structured, and by Day 24 and Day 27, several taxa showed more consistent enrichment or depletion patterns across treatments (Fig. 5; Supplementary Table 6). This progression supports a two-phase interpretation. In the first phase, E-beam irradiation produced dose-associated shifts in the initial community. In the second phase, refrigerated storage favored cold-tolerant and spoilage associated taxa. Ultimately, the highest dose produced the most pronounced early shifts, while storage time remained the dominant factor shaping late microbial composition. These findings suggest that E-beam may improve microbial safety and delay spoilage-associated succession, while storage conditions continue to influence community development throughout the shelf life.

**Fig. 6:**
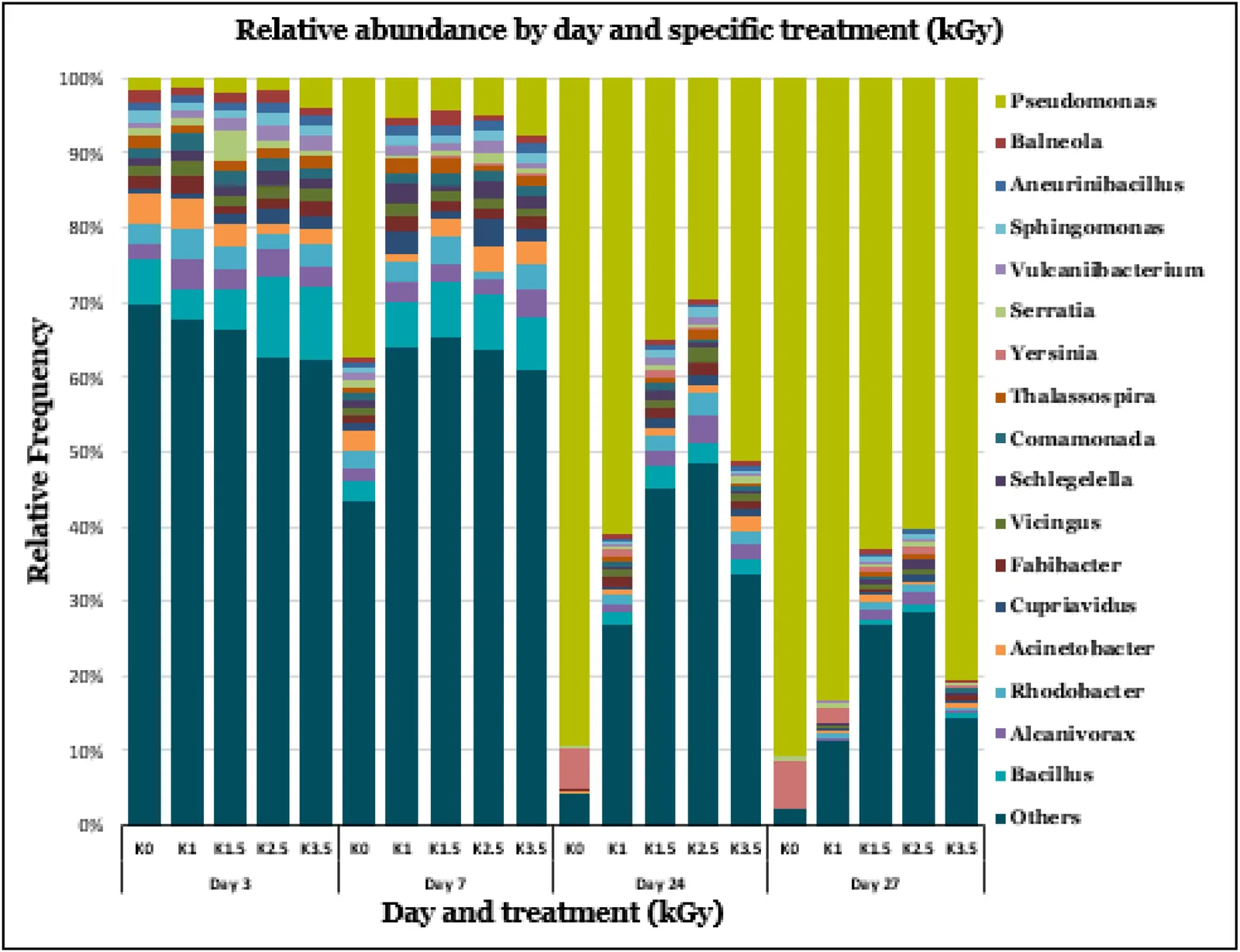
Relative abundance of top 17 dominant bacterial taxa across storage days and electron beam dose treatments. Bars represent microbial community composition for each dose within each sampling day. (Figure generated using excel).

**Fig. 7:**
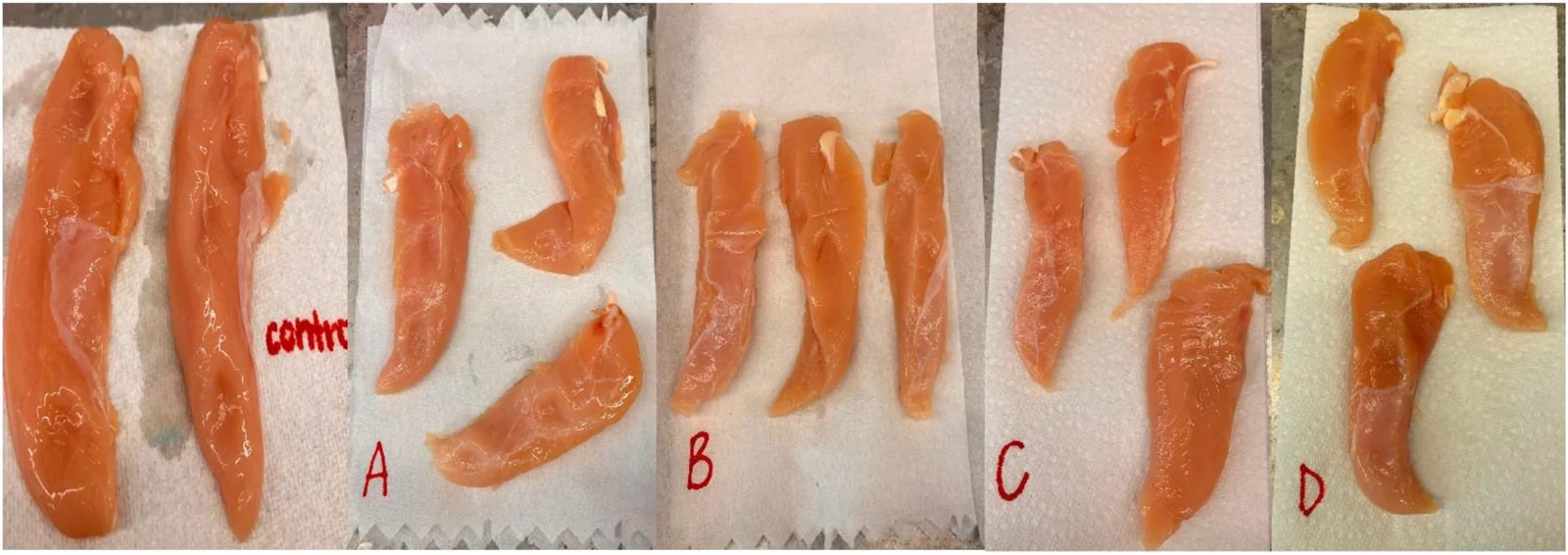
Visual appearance of chicken sausage samples on day 27 of refrigerated storage, comparing the untreated control to samples irradiated at varying doses: (A) 1.0 kGy, (B) 1.5 kGy, (C) 2.5 kGy, and (D) 3.5 kGy. (Figure generated using BioRender).

### *Pseudomonas* enrichment was accompanied by loss of competing and early storage taxa

Cold storage acted as a strong selective pressure favoring psychrotrophic organisms while reducing the relative abundance of taxa less adapted to refrigerated conditions. Early groups, such as *Afipia* and *SAR92_clade* (Supplementary Table 10a and 10b) were among the most enriched genera, but as time progressed these organisms gradually declined. However, these conditions push the community towards a cold adapted and spoilage associated profile dominated by psychrotrophs.

*Pseudomonas* enrichment coincided with a decline in several other taxa, indicating a shift in the relative composition of the microbial community toward lower diversity, and loss of competing microbial groups (Spiers et al., 2000). As *Pseudomonas* became increasingly dominant, the microbial community became progressively less diverse, a pattern consistent with competitive succession during refrigerated storage.

In the current study, chicken tenders were stored in aerobic tray-packed packages. The oxygen availability associated with tray packing may favor the growth of aerobic spoilage organisms such as which *Pseudomonas*. As a result, *Pseudomonas* outcompetes initial colonizers by rapidly exploiting oxygen resources along with available amino acids, simple carbon sources that various other taxa depend on, but cannot defend as effectively (La Rosa et al., 2018). *Pseudomonas* survival mechanism of action is the formulation of biofilms, in which these community of bacteria are enveloped in a matrix of extracellular polymeric substances (EPS) composed mainly of biomolecules, exopolysaccharides, extracellular DNA (eDNA), and polypeptides that forms a hydrated polar mixture (Rasamiravaka et al., 2015). This contributes to the overall structure, potentially giving *Pseudomonas* a competitive advantage and greater access to available nutrients on the meat surfaces (Calhoun et al., 2025). It is important to note that a decline in relative abundance does not necessarily indicate a reduction in absolute population size; rather, the relative increase of *Pseudomonas* may have caused other taxa to become proportionally less abundant. Microbial community diversity changed progressively during refrigerated storage, with reduced diversity observed at later storage stages regardless of the initial E-beam treatment.

By day 24, decreases in *Bacillus* and other early-associated taxa demonstrated their continued presence amongst the depleted groups. These observations are consistent with Faith PD and Shannon diversity, which indicate that community diversity narrows during storage. Furthermore, the declines in *Yersinia, Carnobacterium, Aeromonas, Shewanella, and Brochothrix* (Supplementary Table 3a and 3b). This suggests that late-stage storage does not result in a uniform increase in all spoilage-associated bacteria. Although some of these taxa are commonly associated with meat spoilage or cold storage, their abundance declined in the treated samples.

This pattern suggests that storage conditions eventually select for organisms that can optimally adapt to utilize the available resources for activation of specific metabolic pathways which maximize growth efficiency (La Rosa et al., 2018). As nutrient levels decline while oxygen remains available at the meat surface, the microbial community transitions from a diverse group of early colonizers to a group of species capable of surviving in cold, oxygen, and nutrient-limited environments (Gralka et al., 2020; Ni et al., 2023). As time progresses, the community exhibits reduced diversity and increased specialization, with psychrotrophic and oxidative stress tolerant organisms such as *Pseudomonas* which may become more prominent due to their adaptation to refrigerated storage conditions (Chauhan et al., 2023). These findings suggest that E-beam treatment and storage conditions may alter competitive dynamics, rather than uniformly promoting the growth of all psychrotrophic bacteria.

### Microbial succession and implications for poultry meat spoilage following E-beam irradiation

Poultry meat spoilage is primarily driven by core mechanisms such as microbial growth, autolytic enzymatic activity, and lipid oxidation (Pellissery et al., 2020). The current study contributes to this discussion by showing that microbial community trajectory may be an additional endpoint for evaluating E-beam-treated poultry. Most assessments of E‑beam treatment have emphasized pathogen reduction or total microbial load, yet these measures do not fully capture the impact of irradiation on spoilage ecology. Microbial succession is one component of poultry spoilage, interacting with processes such as autolytic enzymatic activity and lipid oxidation (Luong et al., 2020; Zhu et al., 2022). Therefore, microbial community composition should be evaluated alongside functional measures of product deterioration.

In this study, E-beam treatment influenced microbial succession, including changes in taxa commonly associated with refrigerated meat spoilage. These findings indicate that E-beam irradiation can substantially shape the initial microbial community of tray-packed poultry products. The ecological observation correlates with our previous culture-based findings, in which Collie et al. (2026) demonstrated that E-beam irradiation reduced aerobic and Enterobacteriaceae populations regardless of dosage. Extended refrigerated storage is a primary driver of microbial succession, even following irradiation treatment. Consequently, storage duration, packaging conditions, and cold chain management are critical factors influencing the final microbial composition (Chen et al., 2024; Wang et al., 2024). These findings highlight the necessity to incorporate microbial community succession alongside sensory profiling for a holistic shelf-life understanding.

These patterns also suggest potential strategies for optimizing processing conditions. For instance, while irradiation can reduce certain organisms, refrigerated storage conditions may subsequently favor the persistence and growth of certain taxa. Processors could address this through complementary strategies, such as optimizing the packaging atmosphere to further manage microbial development during storage. Furthermore, identifying microorganisms that persist following irradiation can inform dose optimization and the development of targeted processing strategies for different poultry products.

### Limitations and future directions

A limitation of this study is that 16S rRNA sequencing provides relative abundance data rather than directly quantifying viable bacterial load. Therefore, an increase in the relative abundance of *Pseudomonas* does not necessarily indicate an absolute increase unless supported by culture-based counts or quantitative molecular methods. Future studies should combine 16S sequencing with pathogenic bacteria quantification, psychrotrophic bacteria quantification and *Pseudomonas*-specific enumeration. A second limitation is that the current analysis describes taxonomic succession but does not directly measure spoilage metabolites or sensory outcomes. Since spoilage is a functional process, future work should integrate microbial community profiling with metabolomics, volatile organic compound analysis, pH, purge loss, odor, color, and sensory evaluation (Katiyo et al., 2020). This would help determine whether the observed *Pseudomonas* expansion corresponds to measurable quality deterioration.

Finally, future studies should evaluate whether packaging type, oxygen availability, and cold-chain variation modify the microbial dynamics of E-beam dose. Since *Pseudomonas* is strongly associated with chilled aerobic meat spoilage (Russell, 2000), the packaging atmosphere may influence the extent to which refrigerated storage favors *Pseudomonas* and or other spoilage communities following irradiation. Evaluating these factors together would provide a more complete understanding of how irradiation, packaging, and storage conditions interact to influence poultry shelf life.

## Conclusion

Electron-beam irradiation influenced the microbial ecology of tray-packed chicken tenderloins by shaping the initial community structure and influencing the trajectory of bacterial succession during refrigerated storage. However, storage duration emerged as the primary factor driving long-term community changes, resulting in decreased microbial diversity and a predominance of *Pseudomonas* in later storage stages. The results of the study suggest that E-beam treatment can effectively shape the initial poultry microbiome, while extended refrigerated storage influences the composition of the surviving microbial community. Although E-beam treatment demonstrated effective microbial control, further optimization of processing conditions may help maximize its benefits for poultry meat shelf life. Future work should pair microbial community profiling with viable counts, spoilage metabolite analysis, and meat quality measurements to determine how E-beam-induced microbial shifts contribute to meaningful shelf-life extension.

## Author Contributions

**Blesseth McDonald:** Developed experimental method design. Performed microbiome analysis, drafted and revised the manuscript, approved the final version to be sent for publication. The author has taken full accountability of their efforts and the manuscript. **Elena Olson:** Assisted with data analysis of microbiome data, provided technical guidance, revised the manuscript with additions to writing and approved the final version of the manuscript. The author has taken full accountability of their efforts and the manuscript. **Pheron Collie:** Assisted with data origination, manuscript addition and revision, final revision and approval of the manuscript and has taken full accountability for work. **Gabriela K. Betancourt-Barszcz:** Helped develop the experimental design of this study, performed methodology and contributed with implementation of the experimental procedure, revised and edited the manuscript while approving the final version of the paper. The author has taken full accountability of their efforts and the manuscript. **Olivia Hawkins:** Provided technical analysis and data collection on this project. Revised the manuscript. The author has taken full accountability of their efforts and the manuscript. **Aaron R. Bodie:** Perceived and supervised the project, provided conceptual and technical analysis, assisted with interpretation of the findings, reviewed and revised manuscript and final submission. The author has taken full accountability of their efforts and the manuscript.

## Disclosures

One author (Gabriela K. Betancourt-Barszcz) is employed at the company that operates the electron-beam irradiation device. All authors except for G.K Betancourt-Barszcz declare that this research was conducted without any commercial relationships that could produce potential conflicts of interest.

## Footnotes

Supplementary material associated with this article can be found, in the online version, at doi:10.1016/j.psj.2026.107685.
