# Supplementary material for: Impact of electron-beam dose on microbial diversity and succession dynamics of refrigerated poultry meat during extended storage
Source: Poult Sci. 2026 Aug 22;105(12):107685. doi: 10.1016/j.psj.2026.107685 (PMC13594813; doi:10.1016/j.psj.2026.107685)
Supplement: Supplementary file 1 [file mmc1.docx]

**Tables**

Supplementary table 1. Effect and interaction of treatment and day on the Alpha diversity.

|  |  | **Shannon’s Entropy** | | | **Faith PD** | | | | **Observed features** | | | |
| --- | --- | --- | --- | --- | --- | --- | --- | --- | --- | --- | --- | --- |
|  | DF | SS | F-value | P-value | DF | SS | F-value | P-value | DF | SS | F-value | P-value |
| Treatment | 3 | 0.27 | 0.56 | 0.64 | 4 | 832.91 | 4.86 | **0.003** | 4 | 6991.87 | 0.92 | 0.46 |
| Day | 4 | 2.13 | 3.28 | **0.03** | 1 | 1565.64 | 36.57 | **0.000001** | 1 | 15778.49 | 8.37 | **0.006** |
| Treatment × Day | 12 | 6.43 | 3.30 | **0.006** | 4 | 307.49 | 1.79 | 0.15 | 4 | 11312.24 | 1.50 | 0.22 |
| Residual | 24 | 3.90 |  |  | 31 | 1326.89 |  |  | 31 | 583884.11 |  |  |

(a) Degrees of freedom- DF
(b) Sums of Squares- SS
(c) Bold values represent significant differences

Supplementary table 2. Pairwise comparisons of Alpha diversity metrics across treatment days.

|  |  | **Shannon’s Entropy** | | | **Faith PD** | | | **Observed features** | | |
| --- | --- | --- | --- | --- | --- | --- | --- | --- | --- | --- |
| Group 1* | Group 2 * | H | P-value | Q-value | H | P-value | Q-value | H | P-value | Q-value |
| D0 | D3 | 0.11 | 0.73 | 0.88 | 3.58 | 0.05 | **0.004** | 0.49 | 0.48 | 0.48 |
|  | D24 | 0.00 | 1.00 | 1.00 | 10.29 | **0.001** | **0.004** | 1.34 | 0.24 | 0.29 |
|  | D27 | 2.20 | 0.13 | 0.41 | 11.45 | **0.0007** | 0.05 | 6.12 | **0.01** | 0.05 |
| D24 | D3 | 0.18 | 0.66 | 0.88 | 4.11 | **0.042** | 0.05 | 2.54 | 0.11 | 0.18 |
|  | D27 | 1.06 | 0.30 | 0.60 | 4.26 | **0.038** | 0.05 | 2.40 | 0.12 | 0.18 |
| D27 | D3 | 3.39 | 0.06 | 0.39 | 8.37 | **0.003** | **0.007** | 5.61 | **0.01** | 0.05 |

(a) Bold values represent significant differences
(b) * D: Day

Supplementary table 3. Pairwise comparisons of Alpha diversity metrics across treatments kGy.

|  |  | **Shannon’s Entropy** | | | **Faith PD** | | |
| --- | --- | --- | --- | --- | --- | --- | --- |
| Group 1 | Group 2* | H^2^ | P-value | Q-value | H^2^ | P-value | Q-value |
| K0^a^ | K1 | 0.26 | 0.60 | 0.73 | 1.66 | 0.19 | 0.32 |
|  | K1.5 | 3.01 | 0.08 | 0.41 | 0.10 | 0.74 | 0.74 |
|  | K2.5 | 0.92 | 0.33 | 0.67 | 1.25 | 0.26 | 0.32 |
|  | K3.5 | 4.04 | **0.04** | 0.41 | 5.81 | **0.01** | 0.07 |
| K1^b^ | K1.5 | 1.54 | 0.21 | 0.53 | 0.12 | 0.77 | 0.74 |
|  | K2.5 | 0.26 | 0.60 | 0.73 | 2.01 | 0.15 | 0.31 |
|  | K3.5 | 1.97 | 0.16 | 0.53 | 3.00 | 0.08 | 0.20 |
| K1.5^c^ | K2.5 | 0.18 | 0.66 | 0.73 | 1.42 | 0.23 | 0.32 |
|  | K3.5 | 0.60 | 0.43 | 0.73 | 3.61 | 0.05 | 0.19 |
| K2.5^d^ | K3.5^e^ | 0.00 | 1.00 | 1.00 | 6.31 | **0.01** | 0.07 |

(a) Bold values represent significant differences
(b) ^a^ Control: no kilo gray

^b^ Treatment: Dose of 1 kilo gray	^c^ Treatment: Dose of 1.5 kilo grays

^d^ Treatment: Dose of 2.5 kilo grays

^e^ Treatment: Dose of 3.5 kilo grays*

**Supplementary table 4**: Pairwise comparison for each sample day and treatment based on Faith PD for alpha metrics

|  |  | **Faith PD** | | | | | | | | | | | |
| --- | --- | --- | --- | --- | --- | --- | --- | --- | --- | --- | --- | --- | --- |
|  |  | **Day 0** | | | **Day 3** | | | **Day 24** | | | **Day 27** | | |
| Group 1 | Group 2* | H^2^ | P-value | Q-value | H^2^ | P-value | Q-value | H^2^ | P-value | Q-value | H^2^ | P-value | Q-value |
| K0^a^ | K1 | 0.42 | 0.51 | 0.56 | **0.00** | 1.00 | 1.00 | 3.00 | 0.08 | 0.13 | 3.85 | **0.04** | 0.12 |
|  | K1.5 | 0.04 | 0.82 | 0.82 | 0.19 | 0.27 | 0.66 | 3.85 | **0.04** | 0.13 | 3.85 | **0.04** | 0.12 |
|  | K2.5 | 3.85 | 0.04 | 0.16 | 0.33 | 0.56 | 0.80 | 3.00 | 0.08 | 0.13 | 3.85 | **0.04** | 0.12 |
|  | K3.5 | 3.85 | **0.04** | 0.65 | **0.04** | 0.82 | 1.00 | 3.85 | **0.04** | 0.13 | 3.85 | **0.04** | 0.12 |
| K1^b^ | K1.5 | 0.42 | 0.51 | 0.56 | 1.33 | 0.24 | 0.68 | 0.33 | 0.56 | 0.56 | **0.04** | 0.82 | 0.82 |
|  | K2.5 | 2.33 | 0.12 | 0.25 | 0.60 | 0.43 | 0.80 | 2.40 | 0.12 | 0.15 | **0.04** | 0.82 | 0.82 |
|  | K3.5 | 1.19 | 0.27 | 0.39 | **0.00** | 1.00 | 1.00 | 3.00 | 0.08 | 0.13 | 0.42 | 0.51 | 0.82 |
| K1.5^c^ | K2.5 | 2.33 | 0.12 | 0.25 | 0.33 | 0.56 | 0.80 | 3.00 | 0.08 | 0.13 | 0.42 | 0.51 | 0.82 |
|  | K3.5 | 1.19 | 0.27 | 0.39 | 2.33 | 0.12 | 0.68 | 2.33 | 0.12 | 0.15 | **0.04** | 0.82 | 0.82 |
| K2.5^d^ | K3.5^e^ | 3.85 | **0.04** | 0.16 | 1.33 | 0.24 | 0.68 | 0.33 | 0.56 | 0.56 | **0.04** | 0.82 | 0.82 |

(a) Bold values represent trending differences
(b) ^a^ Control: K0 no kilo gray

^b^ Treatment: Dose of 1 kilo gray	^c^ Treatment: Dose of 1.5 kilo grays

^d^ Treatment: Dose of 2.5 kilo grays

^e^ Treatment: Dose of 3.5 kilo grays*

**Supplementary table 5**: Interaction of treatment and day on the **Beta diversity** metrics

|  |  | **Bray Curtis** | | | | **Jaccard** | | | | **Unweighted Unifrac** | | | | **Weighted Unifrac** | | | |
| --- | --- | --- | --- | --- | --- | --- | --- | --- | --- | --- | --- | --- | --- | --- | --- | --- | --- |
|  | Df | SS | F M | R | P-value | SS | F M | R | P-value | SS | F M | R | P-value | SS | F M | R | P-value |
| Treatment × Day | 9 | 1.63 | 1.38 | 0.20 | 0.05 | 3.39 | 1.02 | 0.22 | 0.13 | 2.13 | 1.00 | 0.21 | 0.44 | 0.89 | 2.18 | 0.19 | **0.038** |
| Residuals | 24 | 3.14 |  | 0.38 |  | 8.84 |  | 0.58 |  | 5.66 |  | 0.55 |  | 1.09 |  | 0.23 |  |
| Total | 40 | 8.06 |  | 1.00 |  | 15.19 |  | 1.00 |  | 10.15 |  | 1.00 |  | 4.60 |  | 1.00 |  |

(a) Degrees of freedom- DF
(b) Sums of Squares- SS
(c) Bold values represent significant differences
(d) F-model- FM

Supplementary table 6. Pairwise comparisons of Beta diversity metrics across days

|  |  |  | **Unweighted Unifrac** | | **Bray Curtis** | | **Weighted unifrac** | |
| --- | --- | --- | --- | --- | --- | --- | --- | --- |
| Group 1* | Group 2* | N | P-value | Q-value | P-value | Q-value | P-value | Q-value |
| D 3 | D7 | 22 | 0.22 | **0.006** | **0.001** | **0.002** | 0.526 | 0.526 |
|  | D24 | 20 | **0.002** | **0.006** | **0.001** | **0.002** | **0.003** | 0.006 |
|  | D27 | 27 | **0.001** | 0.22 | 0.078 | 0.09 | **0.002** | 0.006 |
| D7 | D24 | 14 | **0.037** | 0.17 | 0.278 | 0.27 | **0.005** | 0.007 |
|  | D27 | 21 | **0.004** | 0.05 | **0.002** | **0.003** | **0.001** | 0.006 |
| D 24 | D27 | 19 | 0.14 | **0.008** | **0.001** | **0.002** | 0.369 | 0.442 |

(a) Bold values represent significant differences
(b) * D: Day

Supplementary table 7. Pairwise comparisons of Beta diversity metrics across treatments

|  |  |  | **Bray- Curtis** | | **Weighted Unifrac** | |
| --- | --- | --- | --- | --- | --- | --- |
| Group 1 | Group 2* | N | P-value | Q-value | P-value | Q-value |
| K0^a^ | K1 | 14 | 0.42 | 0.52 | 0.40 | 0.58 |
|  | K1.5 | 16 | 0.84 | 0.84 | 0.53 | 0.65 |
|  | K2.5 | 12 | 0.13 | 0.45 | 0.22 | 0.40 |
|  | K3.5 | 17 | 0.35 | 0.50 | 0.07 | 0.24 |
| K1^b^ | K1.5 | 18 | 0.19 | 0.49 | 0.58 | 0.65 |
|  | K2.5 | 14 | 0.08 | 0.45 | 0.24 | 0.40 |
|  | K3.5 | 19 | 0.63 | 0.70 | 0.07 | 0.24 |
| K1.5^c^ | K2.5 | 16 | 0.33 | 0.50 | 0.70 | 0.70 |
|  | K3.5 | 21 | 0.33 | 0.50 | 0.09 | 0.24 |
| K2.5^d^ | K3.5^e^ | 17 | 0.10 | 0.45 | **0.01** | 0.10 |

(c) Bold values represent trending differences
(d) ^a^ Control: K0 no kilo gray

^b^ Treatment: Dose of 1 kilo gray

^c^ Treatment: Dose of 1.5 kilo grays

^d^ Treatment: Dose of 2.5 kilo grays

^e^ Treatment: Dose of 3.5 kilo grays*

**Supplementary table 8**. Pairwise comparisons of Beta diversity metrics across sample day and treatments

|  |  | **Weighted Unifrac** | | | | | | | | |
| --- | --- | --- | --- | --- | --- | --- | --- | --- | --- | --- |
|  |  |  | Day 3 | | Day 7 | | Day 24 | | Day 27 | |
| Group 1 | Group 2* | N | P-value | Q-value | P-value | Q-value | P-value | Q-value | P-value | Q-value |
| K0^a^ | K1 | 6 | 0.32 | 0.40 | 0.51 | 0.56 | 0.10 | 0.16 | 0.19 | 0.51 |
|  | K1.5 | 6 | 0.40 | 0.45 | 0.10 | 0.40 | 0.09 | 0.16 | 0.31 | 0.51 |
|  | K2.5 | 6 | 0.11 | 0.21 | 0.22 | 0.43 | 0.10 | 0.16 | 0.10 | 0.49 |
|  | K3.5 | 6 | 0.13 | 0.21 | 0.20 | 0.43 | 0.12 | 0.16 | 0.09 | 0.49 |
| K1^b^ | K1.5 | 6 | 0.71 | 0.71 | 0.10 | 0.40 | 0.10 | 0.16 | 0.46 | 0.65 |
|  | K2.5 | 6 | 0.09 | 0.21 | 1.00 | 1.00 | 0.66 | 0.66 | 0.21 | 0.51 |
|  | K3.5 | 6 | 0.10 | 0.21 | 0.29 | 0.43 | 0.40 | 0.47 | 0.60 | 0.75 |
| K1.5^c^ | K2.5 | 6 | 0.11 | 0.21 | 0.12 | 0.40 | 0.10 | 0.16 | 0.79 | 0.79 |
|  | K3.5 | 6 | 0.18 | 0.25 | 0.30 | 0.43 | 0.10 | 0.16 | 0.68 | 0.76 |
| K2.5^d^ | K3.5^e^ | 6 | 0.10 | 0.21 | 0.41 | 0.51 | 0.42 | 0.47 | 0.30 | 0.51 |

(a) ^a^ Control: K0 no kilo gray

^b^ Treatment: Dose of 1 kilo gray	^c^ Treatment: Dose of 1.5 kilo grays

^d^ Treatment: Dose of 2.5 kilo grays

^e^ Treatment: Dose of 3.5 kilo grays*

**Supplementary table 9**. Pairwise comparisons of **Beta diversity** metrics Bray Curtis across treatments and sample day.

|  |  | **Bray Curtis** | | | | | | | | |
| --- | --- | --- | --- | --- | --- | --- | --- | --- | --- | --- |
|  |  |  | Day 0 | | Day 3 | | Day 24 | | Day 27 | |
| Group 1 | Group 2* | N | P-value | Q-value | P-value | Q-value | P-value | Q-value | P-value | Q-value |
| K0^a^ | K1 | 6 | 0.51 | 0.52 | 0.10 | 0.16 | 0.10 | 0.13 | 0.40 | 0.51 |
|  | K1.5 | 6 | 0.30 | 0.52 | 0.11 | 0.16 | 0.09 | 0.13 | 0.19 | 0.33 |
|  | K2.5 | 6 | 0.52 | 0.52 | 0.09 | 0.16 | 0.10 | 0.13 | 0.09 | 0.26 |
|  | K3.5 | 6 | 0.48 | 0.52 | 0.09 | 0.16 | 0.09 | 0.13 | 0.09 | 0.26 |
| K1^b^ | K1.5 | 6 | 0.50 | 0.52 | 0.10 | 0.16 | 0.09 | 0.13 | 0.51 | 0.57 |
|  | K2.5 | 6 | 0.10 | 0.28 | 0.65 | 0.65 | 0.32 | 0.36 | 0.10 | 0.26 |
|  | K3.5 | 6 | 0.10 | 0.28 | 0.20 | 0.25 | 0.52 | 0.52 | 0.19 | 0.33 |
| K1.5^c^ | K2.5 | 6 | 0.10 | 0.28 | 0.98 | 0.16 | 0.10 | 0.13 | 0.90 | 0.90 |
|  | K3.5 | 6 | 0.11 | 0.28 | 0.10 | 0.16 | 0.07 | 0.13 | 0.28 | 0.41 |
| K2.5^d^ | K3.5^e^ | 6 | 0.31 | 0.52 | 0.28 | 0.33 | 0.10 | 0.13 | 0.09 | 0.26 |

(a) ^a^ Control: K0 no kilo gray

^b^ Treatment: Dose of 1 kilo gray	^c^ Treatment: Dose of 1.5 kilo grays

^d^ Treatment: Dose of 2.5 kilo grays

^e^ Treatment: Dose of 3.5 kilo grays*

Supplementary table 10 a. Relative abundance based on enriched and depleted genera at Day 3 across irradiation treatments.

| **Day 3** | | | | |
| --- | --- | --- | --- | --- |
| **Dose kGy^a^** | **Total Enriched** | **Top Enriched Genera lfc** | **Total Depleted** | **Top Depleted Genera lfc** |
| 1 | 89 | *Bradyrhizobium 4.68 AB‑179‑E04 4.16*  *clade 3.54* | 68 | *NS4_marine_group –3.26 Asinibacterium –3.01*  *uncultured –2.98* |
| 1.5 | 79 | *SAR92_clade 3.49*  *uncultured 3.27 Stenotrophomonas 3.26* | 78 | *Subgroup_2 –4.21*  *Soonwooa –3.73*  *NS9_marine_group –3.62* |
| 2.5 | 73 | *NS2b_marine_group 3.00 Polaribacter 2.92*  *Spiroplasma 2.92* | 84 | *uncultured –4.36*  *NS9_marine_group –3.62*  *unassigned –3.42* |
| 3.5 | 78 | *Rhizobiales 2.76*  *Methanolinea 2.63 Staphylococcus 1.96* | 79 | *Altererythrobacter –4.60*  *Serratia –4.24*  *Subgroup_2 –4.21* |

(A) kGy^a^- Kilograys
(B) lfc^b^- Log fold change

Supplementary table 10 b. Relative abundance based on enriched and depleted genera at Day 7 across irradiation treatments.

| **Day 7** | | | | |
| --- | --- | --- | --- | --- |
| **Dose kGy** | **Total Enriched** | **Top Enriched Genera lfc** | **Total Depleted** | **Top Depleted Genera lfc** |
| 1 | 82 | *uncultured 4.58*  *Afipia 4.55*  *unassigned 4.31* | 77 | *uncultured –4.77*  *Asinibacterium –4.34 Stenotrophomonas –3.25* |
| 1.5 | 66 | *Aminicenantales 4.59*  *Afipia 4.48*  *Unassigned 3.40* | 93 | *SG8‑4 –3.26*  *GWB1‑42‑6 –2.84*  *Nitrosotalea –2.81* |
| 2.5 | 77 | *unassigned 4.39*  *Afipia 4.16 NS4_marine_group 4.05* | 82 | *OM182_clade –3.15*  *unassigned –2.80*  *Subgroup_2 –2.76* |
| 3.5 | 71 | *Afipia 3.11*  *0319‑6G20 2.95*  *uncultured 2.83* | 88 | *PeM15 –4.42*  *Lactobacillus –4.04*  *Cloacibacterium –3.13* |

(A) kGy^a^- Kilograys
(B) lfc^b^- Log fold change

Supplementary table 10 c. Relative abundance based on enriched and depleted genera at day 24 across irradiation treatments.

| **Day 24** | | | | |
| --- | --- | --- | --- | --- |
| **Dose kGy** | **Total Enriched** | **Top Enriched Genera lfc** | **Total Depleted** | **Top Depleted Genera lfc** |
| 1 | 61 | *Bacillus 4.83*  *Alcanivorax 4.69*  *Chloroplast 4.23* | 51 | *Aeromonas –3.62*  *Shewanella –3.37*  *Yersinia –1.81* |
| 1.5 | 48 | *Bacillus 3.67*  *Rhodobacter 3.67 Alcanivorax 3.61* | 63 | *Aeromonas –4.37*  *Shewanella –4.37*  *Carnobacterium –3.27* |
| 2.5 | 59 | *Bacillus 4.52*  *Alcanivorax 4.50 Rhodobacter 4.34* | 53 | *Yersinia –4.83*  *Carnobacterium –3.89*  *Aeromonas –3.80* |
| 3.5 | 44 | *Bacillus 3.35*  *Alcanivorax 3.06*  *uncultured 2.90* | 68 | *Yersinia –8.41*  *Carnobacterium –5.34*  *Shewanella –4.99* |

(A) kGy^a^- Kilograys
(B) lfc^b^- Log fold change

Supplementary table 10 d. Relative abundance based on enriched and depleted genera at Day 27 across irradiation treatments.

| **Day 27** | | | | |
| --- | --- | --- | --- | --- |
| **Dose kGy** | **Total Enriched** | **Top Enriched Genera lfc** | **Total Depleted** | **Top Depleted Genera lfc** |
| 1 | 31 | *Unassigned 4.33 Chloroplast 4.00 Rhodobacter 2.78* | 39 | *Brochothrix –1.29*  *Yersinia –1.04*  *Carnobacterium –0.36* |
| 1.5 | 23 | *unassigned 2.39 Chloroplast 1.91 uncultured 1.59* | 47 | *Yersinia –4.82*  *unassigned –4.18*  *Serratia –3.96* |
| 2.5 | 20 | *unassigned 2.59 Chloroplast 2.59 uncultured 1.63* | 50 | *Yersinia –4.32*  *unassigned –4.00*  *Brochothrix –3.72* |
| 3.5 | 47 | *unassigned 4.74 Chloroplast 4.66 Acinetobacter 3.83* | 23 | *Yersinia –5.53*  *Brochothrix –3.55*  *Carnobacterium –2.70* |

(A) kGy^a^- Kilograys
(B) lfc^b^- Log Fold Change
